# Look who’s talking: Two-mode networks as representations of a topic model of New Zealand parliamentary speeches

**DOI:** 10.1371/journal.pone.0199072

**Published:** 2018-06-20

**Authors:** Ben Curran, Kyle Higham, Elisenda Ortiz, Demival Vasques Filho

**Affiliations:** 1 Te Pūnaha Matatini, University of Auckland, Auckland, New Zealand; 2 Te Pūnaha Matatini, School of Chemical and Physical Sciences, Victoria University of Wellington, Wellington, New Zealand; 3 Te Pūnaha Matatini, School of Engineering and Computer Science, Victoria University of Wellington, Wellington, New Zealand; 4 Department of Physics, The University of Auckland, Auckland, New Zealand; Centre National de la Recherche Scientifique, FRANCE

## Abstract

Quantitative methods to describe the participation to debate of Members of Parliament and the parties they belong to are lacking. Here we propose a new approach that combines topic modeling with complex networks techniques, and use it to characterize the political discourse at the New Zealand Parliament. We implement a *Latent Dirichlet Allocation* model to discover the thematic structure of the government’s digital database of parliamentary speeches, and construct from it *two-mode networks* linking Members of the Parliament to the topics they discuss. Our results show how topic popularity changes over time and allow us to relate the trends followed by political parties in their discourses with specific social, economic and legislative events. Moreover, the community analysis of the two-mode network projections reveals which parties dominate the political debate as well as how much they tend to specialize in a small or large number of topics. Our work demonstrates the benefits of performing quantitative analysis in a domain normally reserved for qualitative approaches, providing an efficient way to measure political activity.

## 1 Introduction

Topic models have received widespread attention in recent years [1–4] as they have proven to be useful tools for dealing with the vast amount of semantic information that is becoming available. Topic modeling encompasses a set of machine learning techniques which take a collection of documents as input and attempt to discern the themes that pervade them [5]. Although the methods that topic models utilize to search, summarize and understand large electronic archives are rapidly popularizing in several areas, they have rarely been applied to political texts.

The New Zealand government has been making parliamentary transcripts (‘Hansard’) available in digital format since 2003. Suitable annotation of these transcripts allow them to be used as a corpus for the development of topic models. This comprehensive corpus of political text can then be examined through a number of lenses. Topic models allow us to monitor the ebb and flow of themes that are discussed in parliament over multiple years and associate particular themes with individual Members of Parliament (MPs). This enables the identification of trends of topics that particular parties follow. That is, we may observe which issues are discussed repeatedly with great interest and which cease to be mentioned.

A number of textual analyses of political speeches are concerned with finding where on the political spectrum a speaker falls [6–8]. Topic modeling as applied in our analyses cannot determine the sentiment of a statement or speech. Despite this fact, we demonstrate that multiple aspects of politicians’ policy interests can be unraveled with further statistical analysis, specially implementing complex networks techniques.

In this work we use the parliamentary speeches data, as classified by a Latent Dirichlet Allocation topic model, to construct two-mode networks [9–12]. These networks consist of a set of MPs which are linked to a set of topics, where each link represents a topic that is of clear interest to a particular MP. Two-mode networks can be more easily analyzed when decomposed into projections, that is, weighted networks which contain only one set of nodes (either MPs or topics). Here we focus on the MP-projection, which is particularly interesting because it constitutes a network where a link between two MPs indicates the existence of a mutual interest. Measuring properties such as the node degree distributions, homophily [13, 14], clustering and community structure [15, 16] of these networks provides important insights into politicians activity. For instance, one can discover whether or not the typical range of interests of an MP is changing, as well as patterns in this behavior over time. Moreover, community-detection methods [17–20] reveal clusters of politicians that share common interests. This allows us to uncover the partisan composition of these communities during the four studied parliamentary terms.

For most of the 20^th^ century, the left-leaning Labour Party and right-leaning National Party have been the two parties sharing power at New Zealand government. However, in 1996, the method of electing MPs was changed to a mixed-member proportional (MMP) system and these two parties were joined by a number of smaller parties, which sometimes have held the balance of power. In fact, the period analyzed includes a transition of power from the 5^th^ Labour government (1999-2008) to the 5^th^ National government (2008-).

This work is of an exploratory nature, in that our goal is twofold: to present a novel quantitative approach of measuring political activity and to demonstrate the benefits of performing quantitative analysis in a domain normally reserved for qualitative approaches, by using a combination of machine learning and complex networks techniques.

The remainder of this paper is organized as follows: the Methods and Data sections 2.2 and 2.3 introduce fundamental aspects of topic modeling and two-mode networks respectively and outline the preparation and organization of our data; Sections 3 and 4 present the results of our analyses alongside a discussion and our conclusions.

## 2 Methods and data

### 2.1 Data

The semantic data we are using for our analyses are extracted from the New Zealand Hansard database [21]. Hansard presents records of what is said in the debating chamber as debates (a collection of speeches on a particular topic), speeches (individual statements by MPs) or dailies and volumes (collections of speeches over different time periods). By considering only those documents labeled in that database as a ‘speech’ delivered between February 2003 and August 2016 we were able to find out in which topics specific MPs were engaging with. This makes it possible to associate speeches and by extension MPs with topics of interest over time.

Once these data are obtained, we observe that many speeches are rather short and contain little topical content. An example is given below, which comes from a committee discussion on the *Shop Trading Hours Amendment Bill* and was published in Hansard Volume 716 on the 17^th^ of August 2016 [21]:

“**CHAIRPERSON (Lindsay Tisch)**: Just a point: this debate concludes at quarter past and to whoever is speaking at the time, I will be stopping it at that point.”

**Fig 1:**
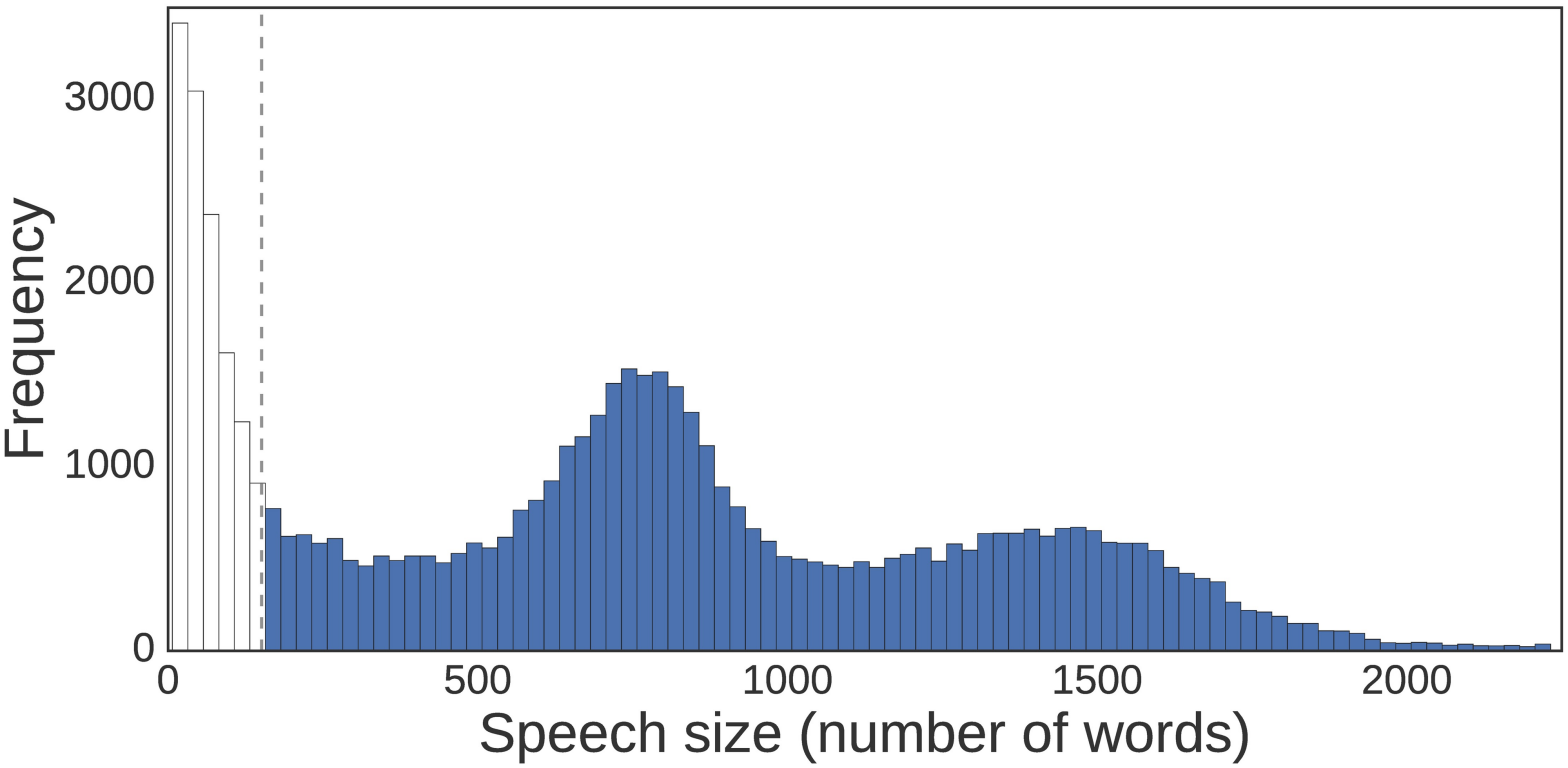
Distribution of word-counts for New Zealand parliamentary speech. Speeches delivered in the period February 2003 to August 2016. In blue, the speech sizes used in our analysis. Speeches shorter than 150 words are omitted from our analysis due to lack of topical content, this threshold is indicated by the dashed vertical line.

In an attempt to remove these non-topical speeches, we have removed from our database speeches with 150 words or less, which constitute about 20% of the database. This cut-off is shown in Fig 1 which presents the distribution of word-counts per speech. This decision is informed by observations of the insufficient topical content of speeches below this threshold.

### 2.2 Topic models

The process of topic modeling involves utilizing a set of algorithms that have been developed to understand the underlying thematic structure of a corpus. The simplest and most commonly used topic model is Latent Dirichlet Allocation (LDA) [22]. Within the framework of LDA, each document is a mixture of corpus-wide topics and each topic can be understood as a distribution over keywords. The total number of documents comprising the corpus is denoted as *D* and the total number of topics as *K*. Additionally, the order of words that comprise the document is not considered, only the frequency with which words appear.

From the perspective of LDA, documents are imagined to be the result of a generative process. This is the process by which the model assumes the documents arose given certain *hidden variables*. The word-distributions per document are observed, while the topic structure—per-word topic assignment, per-document topic proportions and per-corpus topic distributions—are hidden elements. Therefore, the central computational problem for LDA is to infer the hidden structure that likely generated the observed corpus. This means computing the conditional distribution of the hidden variables given what is observed. This conditional distribution is usually referred to as the *posterior* and can be expressed as

$$\begin{array}{c} p\left(\beta_{1:K},\theta_{1:D},z_{1:D}|w_{1:D}\right)=\frac{p(\beta_{1:K},\theta_{1:D},z_{1:D},w_{1:D})}{p(w_{1:D})} \end{array}$$ (1)

where *β*_1:*K*_ are all topics in the corpus, *θ*_1:*D*_ are the per-corpus topic proportions, *z*_1:*D*_ the per-corpus topic assignments and *w*_1:*D*_ the whole set of observed words. Unfortunately, computing the posterior is computationally unfeasible and hence needs to be approximated by an inference algorithm. Consequently, topic modeling algorithms are commonly classified as sampling-based algorithms or variational algorithms [23].

In this work, we used the MALLET software package [24] for the topic modeling component of our analysis. MALLET implements Gibbs sampling [25], which constructs a sequence of random variables in a Markov chain, where each variable is dependent on the previous. The algorithm then assumes that the true posterior distribution is the limiting distribution of this sequence, and obtains an approximation to this posterior using these samples. For a full mathematical description of LDA and a further discussion of the methods used to estimate a posterior, see [23].

**Fig 2:**
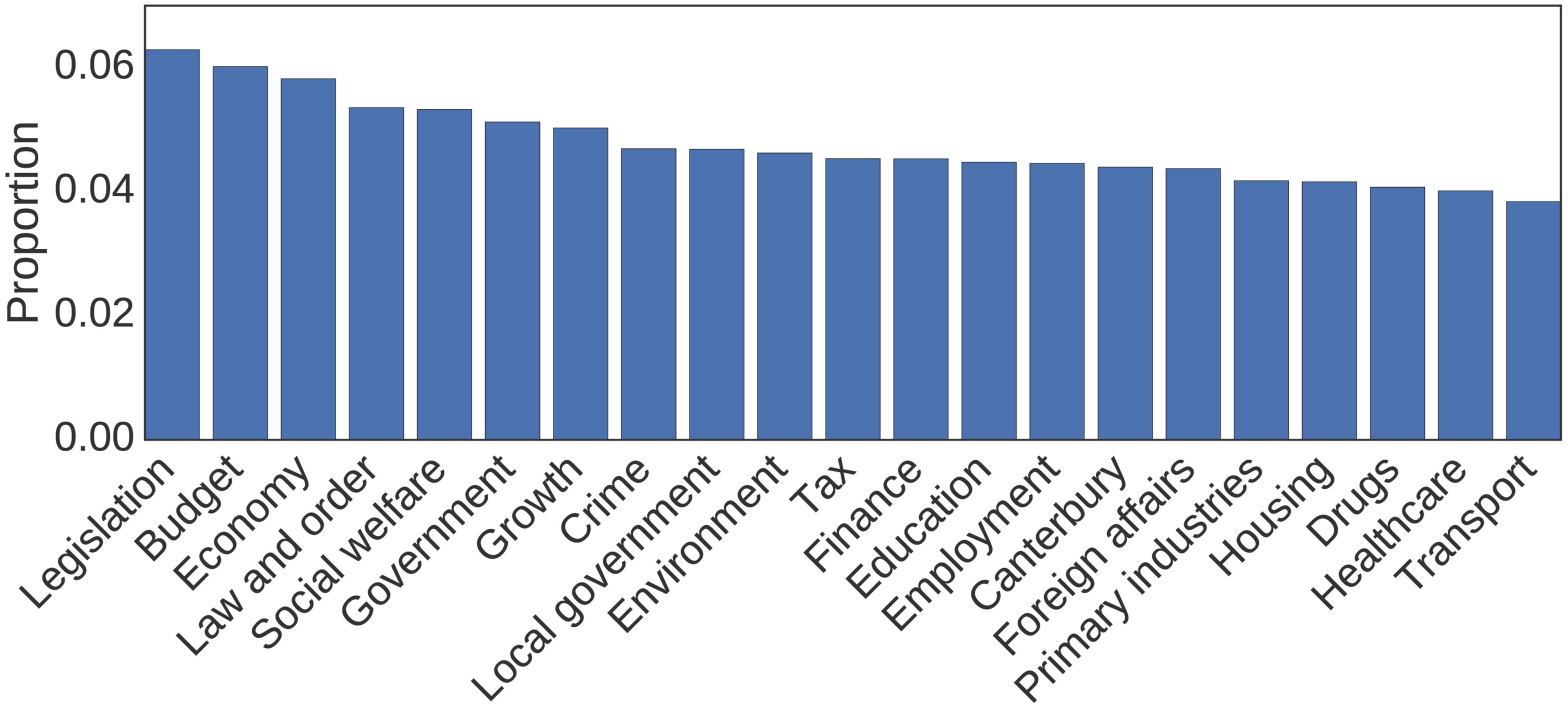
Topics identified by an LDA-based topic model of the Hansard speech corpus. Topics for the period February 2003 to August 2016 and their respective normalized proportions as a fraction of all the topical content discovered after the omission of nine topics with little specific thematic content. A list of topics and the keywords that comprise them can be found in S2 Table.

LDA assumes the topics are the same for all documents, and only the topic proportions vary. Therefore, MALLET requires an input which specifies the number of topics to be discovered. Choosing this number is critical to the success of a topic model, as too few topics may merge distinct themes, while too many topics may introduce many “themes” consisting of vocabularies that appear to have nothing in common, or even start splitting topics that were identifiable at smaller input values. For our analysis it is important that the topics are easily identifiable and distinct from one another. We found that 30 topics satisfies these requirements. Identified topics and their corresponding keywords can be found in S2 Table. It is worth noting, however, that some topics (nine of them, corresponding to about 36% of the corpus) appeared to consist mainly of terms that were primarily either procedural or general rhetoric, such as “proud”, “hope” or “nation”. As this language reveals little in the way of substantive interactions, such topics were omitted from our subsequent analyses after networks had been inferred. Fig 2 shows the remaining topics with their rescaled proportions.

### 2.3 Two-mode networks

A great number of real world networks are, in fact, one-mode projections of bipartite architectures comprised of two different types of nodes. Such architectures are called two-mode networks, also known as bipartite networks. A two-mode network is, therefore, mathematically defined as a bipartite graph *G* = {*U*, *V*, *E*}, where *U* and *V* are disjoint sets of nodes and *E* = {(*u*_*i*_, *v*_*j*_): *u*_*i*_ ∈ *U*, *v*_*j*_ ∈ *V*} is the set of links connecting these nodes. For our purposes, the sets *U* and *V* correspond to the sets of MPs and topics, and the set *E* represents the links that emerge when an MP speaks sufficiently frequently about a topic. No connections among nodes of the same set are allowed in the bipartite network, that is, MPs are connected only to topics not other MPs and vice versa. Each set of nodes can have independent properties, such as the degree distributions, or the number of nodes (system size).

Once we find the set of topics that a particular MP speaks about ‘often’ enough (this criterion is defined below), these are represented as links between the MP and those topics. After this process is completed for all MPs, we can construct a two-mode network where nodes representing MPs are connected only to nodes representing topics, and vice versa.

Bipartite structures play an important role in the analysis of social and economic networks. They are normally used to represent conceptual relations—such as membership, affiliation, collaboration, employment, ownership and others—between two different types of entities within a system [10–12]. Often, we are more interested in one of the types of nodes (e.g. MPs) and, in order to investigate the relationships between them, we create a new network with only these nodes. This new network is a *projection* of the original two-mode network.

**Fig 3:**
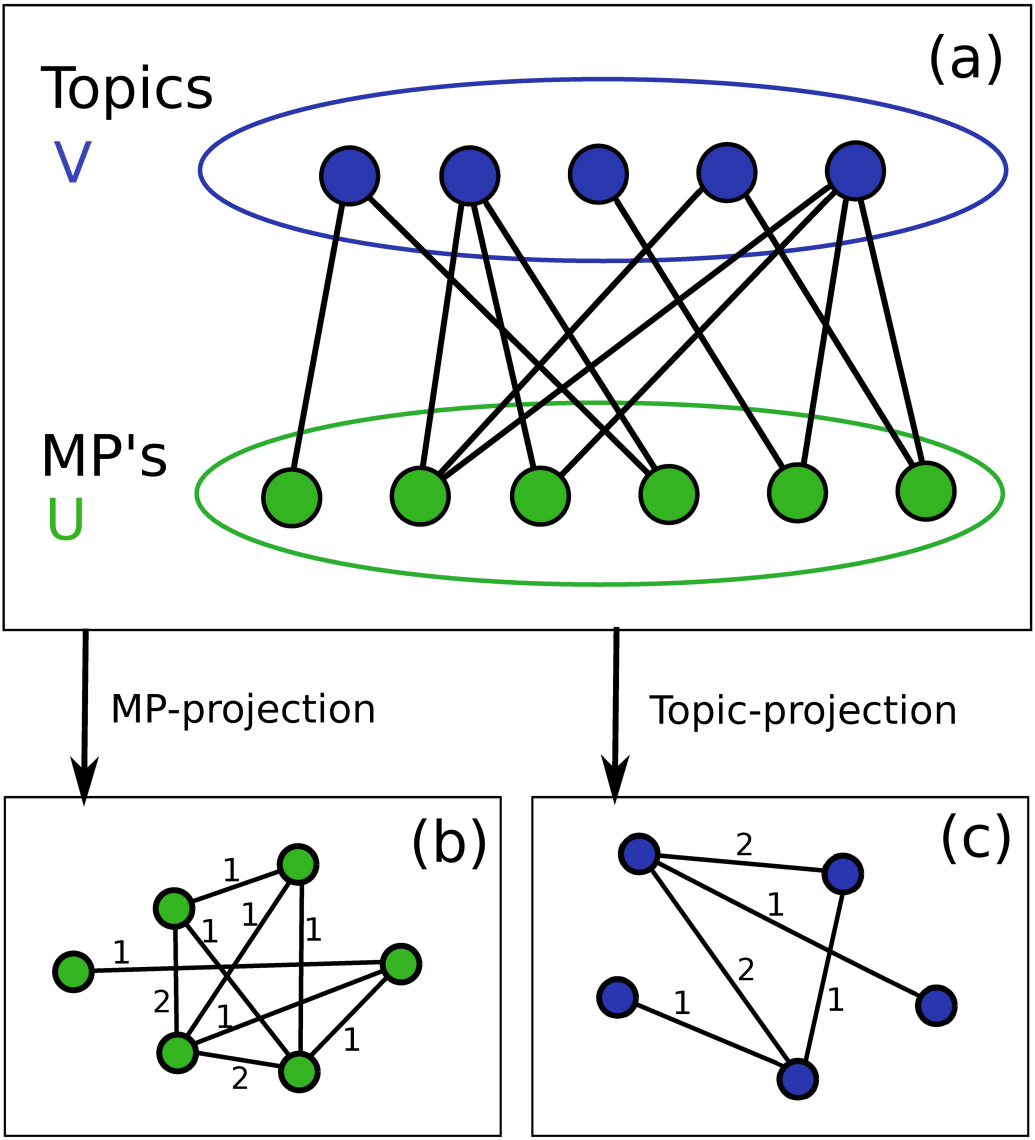
Schematic drawing of a bipartite network and its projections. **(a)** Bipartite network and its projections onto set of nodes **(b)** *U*, where nodes represent MPs, and **(c)** *V*, where nodes represent topics. In (b) and (c) the numbers on top of the links indicate the weights, which correspond to the number of common neighbors that two linked nodes share in the bipartite network.

Topic modeling results in a natural bipartite network with projections that can be easily interpreted. The projections of a bipartite network are obtained by connecting nodes which share a common neighbor. That is, if two MPs are both linked to the same topic in the bipartite network, then they are linked in the MP-projection. For a bipartite network, this process results in two completely separated components, each composed exclusively of one type of node (MPs and topics in our case). Fig 3 shows a schematic drawing of a bipartite network and its possible projections. The edges between nodes in these projections are then weighted, dependent on the number of neighbors the nodes share in the bipartite network. In our analyses we use *simple weighting* method [26], whereby each edge has a weight that equals the number of neighbors the nodes share in the bipartite network. If two MPs are linked to the same three topics in the bipartite network, then the edge linking them in the MP-projection will have weight equal to three.

The weighting in these projections offers a way to eliminate edges that represent tenuous links. This is important, as *complete subgraphs* (where every node is connected to every other node in the subgraph) of MPs are generated by every topic. This means that every MP that speaks about a popular topic is connected to every other MP that speaks about that same topic. The existence of popular topics can make analyses such as community detection challenging in the absence of weighting.

In order to build bipartite networks connecting MPs to topics, we looked at the corpus of each MPs speeches in more detail. We considered an MP to be connected to a topic when at least 6.7% of the MP’s speeches over the course of a year was assigned to that topic by MALLET. This occurs when MPs talk about a topic twice as much as would be expected if they were talking about all topics equally within a year. This method, removes topics that MPs only touch on briefly or in passing, which does not indicate engagement with the topic. Finally, MPs that had spoken less than 10^4^ words in the entire term were removed form the network for the lack of significance in the volume of words spoken.

## 3 Results

### 3.1 Words spoken

**Fig 4:**
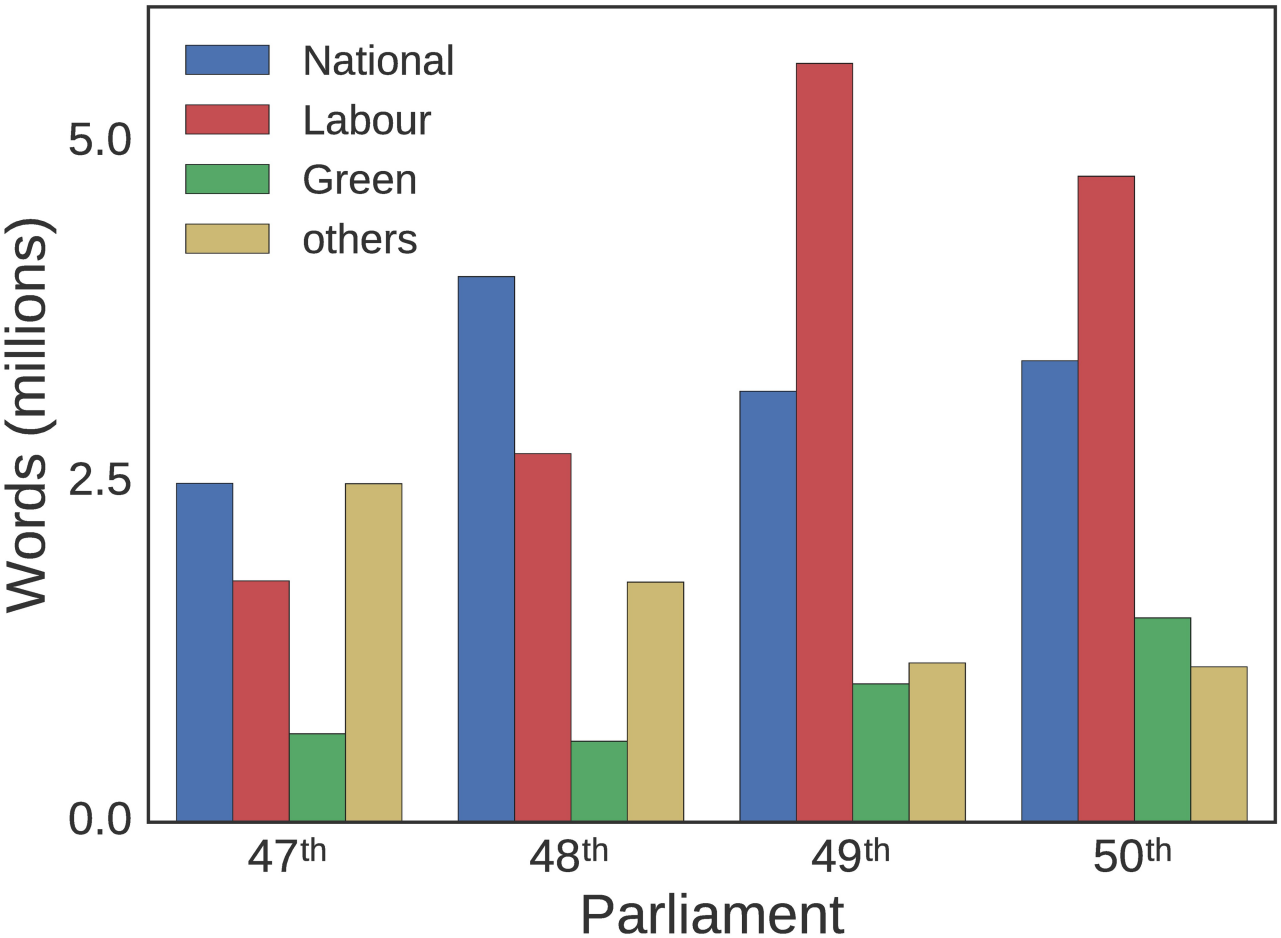
Total number of words spoken by each party. Number of words in their speeches longer than 150 words, for each of the four complete parliamentary terms studied.

Despite having fewer MPs, opposition parties tend to have a greater total word count than the governing party. Fig 4 shows the total word count for each of the 3 largest parties (as of the 50th parliament) over the course of 4 parliaments. In each parliament, the total word count for opposition parties exceeds that of the governing party. This is likely due to opposition MPs attempting to draw attention to what they see as the governing parties shortcomings [27]. The increase in words spoken does not appear to be driven by any particular MP or small group of MPs (S2 Fig).

### 3.2 Time series of topic popularity

**Fig 5:**
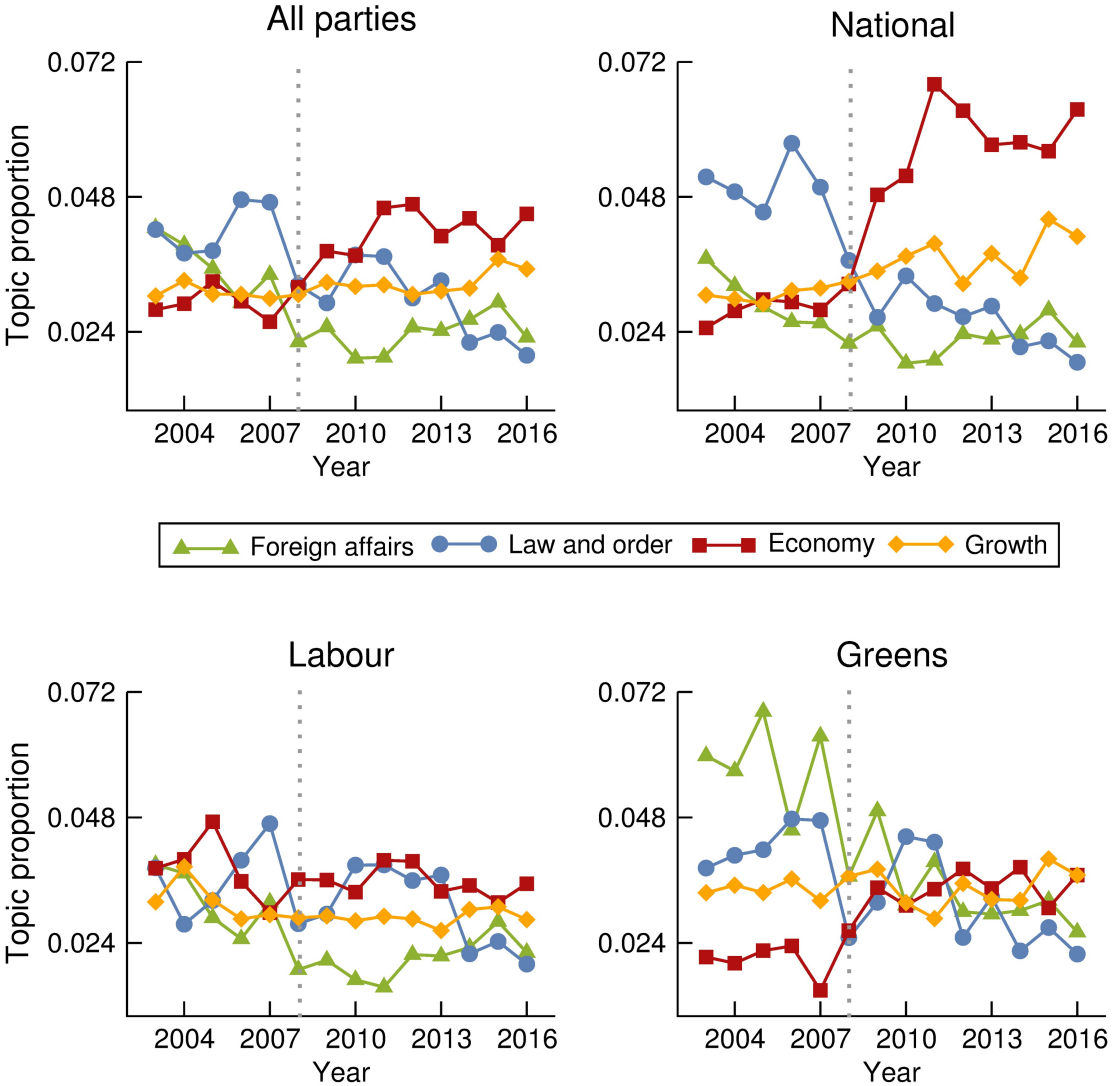
Evolution of topic proportions 1. Evolution over time of the proportions in which the topics labeled as *Foreign Affairs*, *Law and order*, *Economy*, and *Growth*, are discussed at the Parliament, and the corresponding decomposition by party. Grey dotted lines indicate the change of government from Labour to National in 2008.

**Fig 6:**
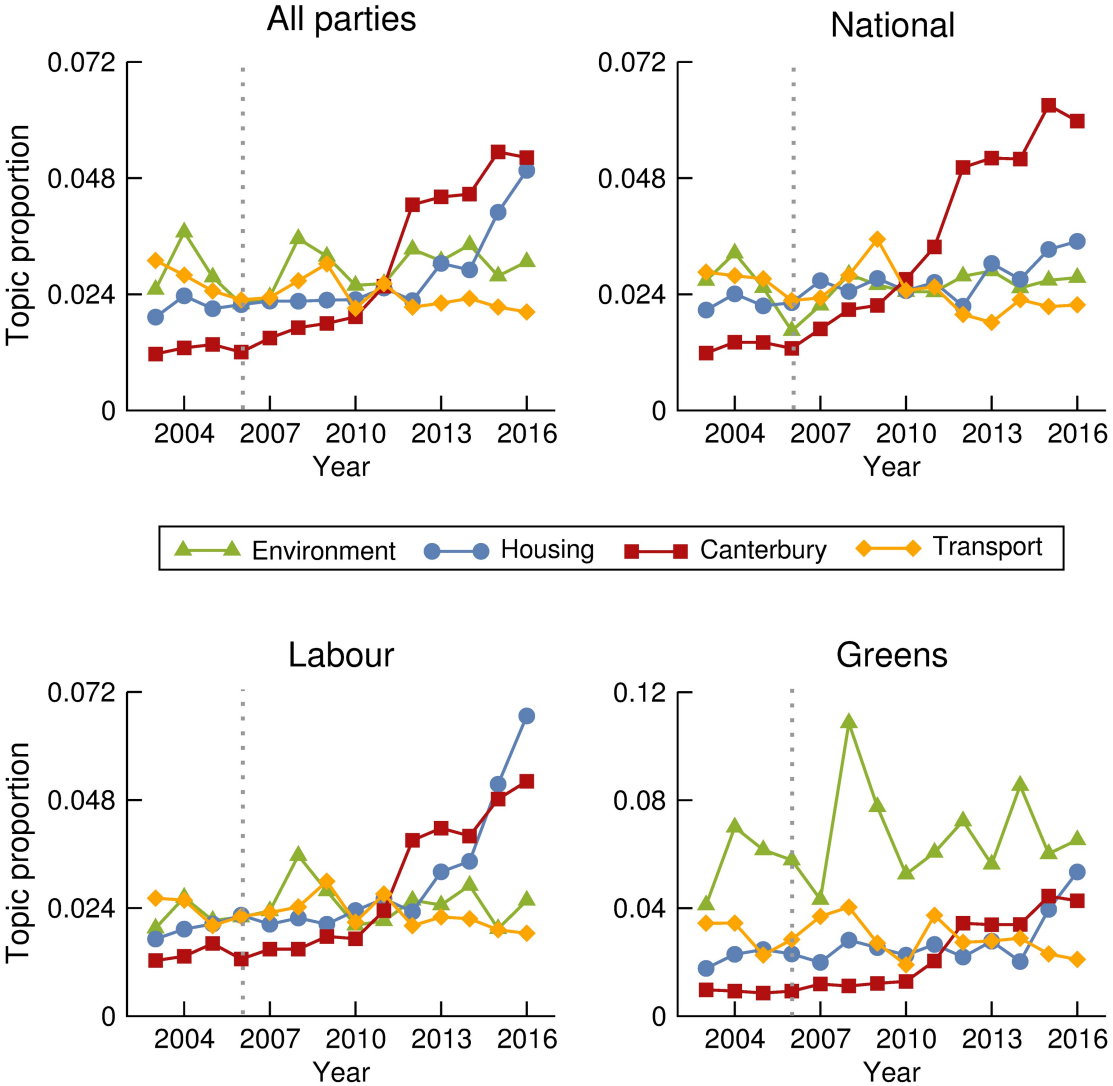
Evolution of topic proportions 2. Evolution over time of the proportions in which the topics labeled as *Environment*, *Housing*, *Canterbury*, and *Transport*, are discussed at the Parliament, and the corresponding decomposition by party. Grey dotted lines indicate the change of government from Labour to National in 2008. Note the change in scale in the Topic proportion axis for the Green Party time series to accommodate their notable interest in environmental policy.

Allowing a decomposition by party, we ran a topic model on data concatenated by MP and year. The topic proportions obtained over a total number of 30 topics are normalized for each year so that they are comparable across a time span of 14 years. Proceeding this way, we can reproduce the evolution of topic popularity over time at the Parliament and its decomposition for each of the three most represented parties. Clear trends and differences across parties are visible in Figs 5 and 6. Evolution of proportion of other argued topics appears in S1 Fig.

Changes in topics being discussed often correlate with real world events such as the financial crisis of 2008 (Fig 6), Canterbury and the Christchurch earthquakes of 2010/2011 (Fig 5) and more recently the housing crisis from about 2013 (Fig 5).

### 3.3 The parliamentary speech network

**Fig 7:**
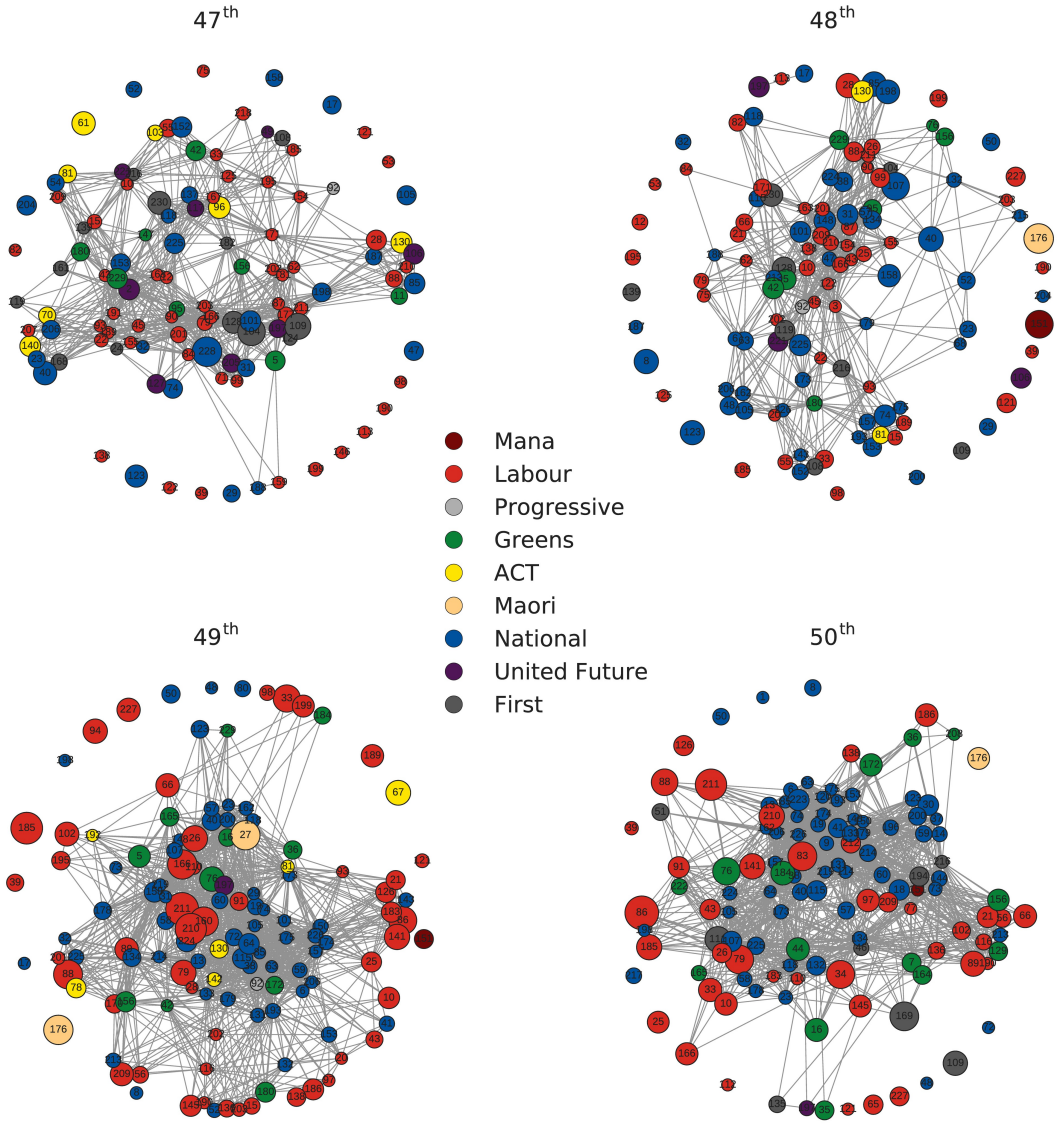
MP networks for parliaments 47^th^ to 50^th^. Networks corresponding to the MP-projection of the original bipartite network. Node size is proportional to the total number of words spoken by each MP over the course of the parliamentary term. Node labels identifying MPs are provided in S1 Table.

The MP-projected networks for the 47^th^ to 50^th^ parliaments resulting from the process described above are shown in Fig 7. The community structure [20] in these networks is visible, as is the party make-up of these communities. Table 1 shows the number of MPs per party present in each of these four networks.

**Table 1:** Number of members of parliament in the three largest parties (as of the 50^th^ parliament) for each complete parliamentary term studied.

Term	National	Labour	Green	Others
47^th^	26	52	8	29
48^th^	48	50	7	16
49^th^	58	47	11	11
50^th^	61	39	14	11

Before examining the structure of the network, it is important to note that in all networks a number of MPs are not connected to any others. This is a reflection of our methodology. The 6.7% threshold that is applied to filter topics may in fact remove all topics in the unlikely scenario that the MP in question talks about many topics in roughly equal proportion. MPs with diverse interests may not be identified as fitting into any particular community.

**Fig 8:**
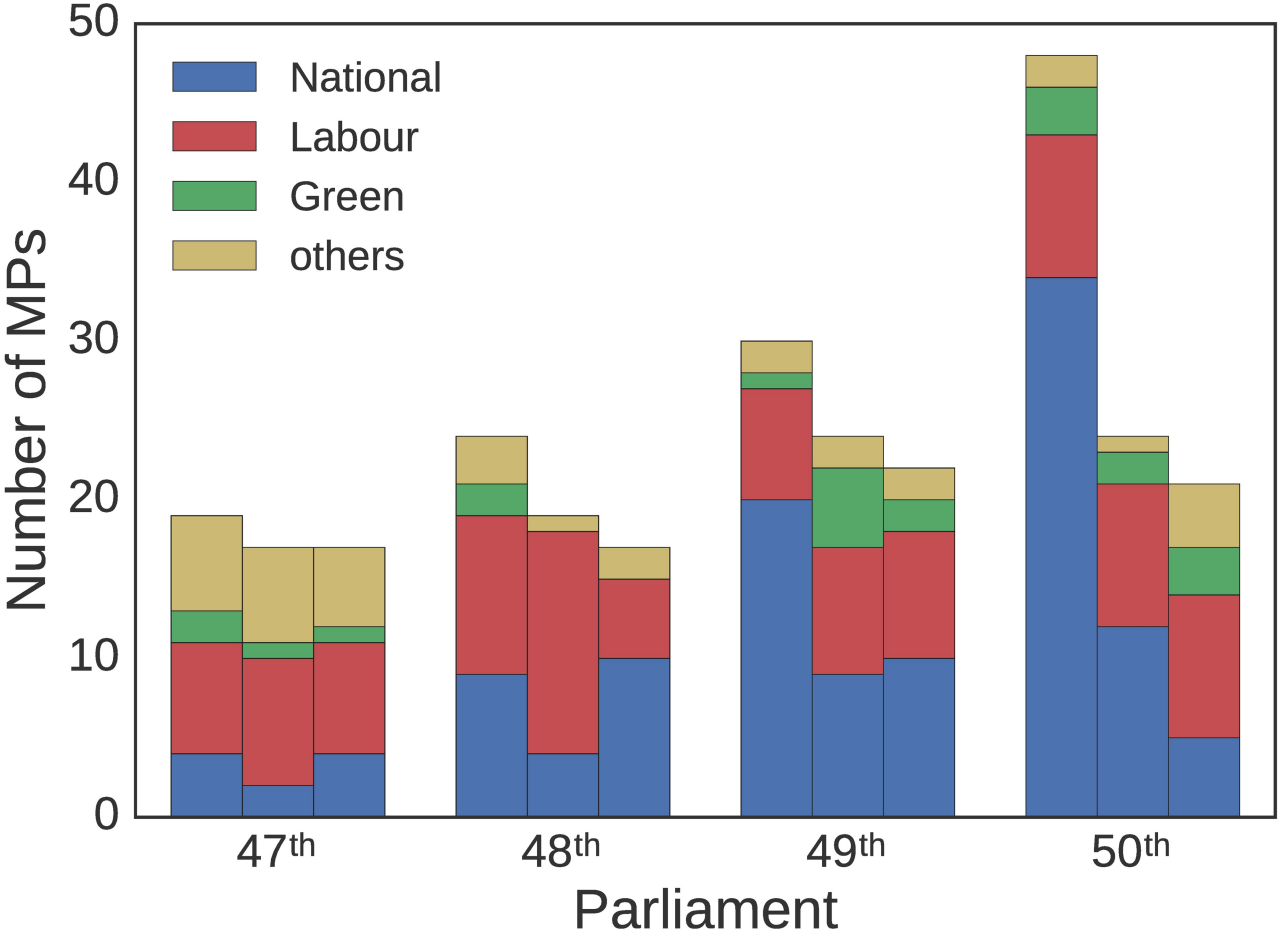
Size and party composition of the three largest communities. Network communities discovered in the MP-projected network for each of the parliaments examined.

A striking difference between the MP networks of the first two parliaments and last two is the party composition of visible communities. In the first two (Labour government, 2002-2008) the communities are quite party-wise diverse, while in the last two (National government, 2008-2014) there is a close-knit community made up of National Party MPs. This is corroborated by the three largest communities composition, for every term in our analysis, shown in Fig 8 [17]. In the first two terms, we note heterogeneous, smaller core communities and the absence of a community that is much larger than the others. That changes for the last two terms, specially the last one, where we observe one community much larger than the rest emerging, dominated by MPs of the National Party. Another point worth noticing is the smaller presence of minor parties in the largest communities over time.

**Fig 9:**
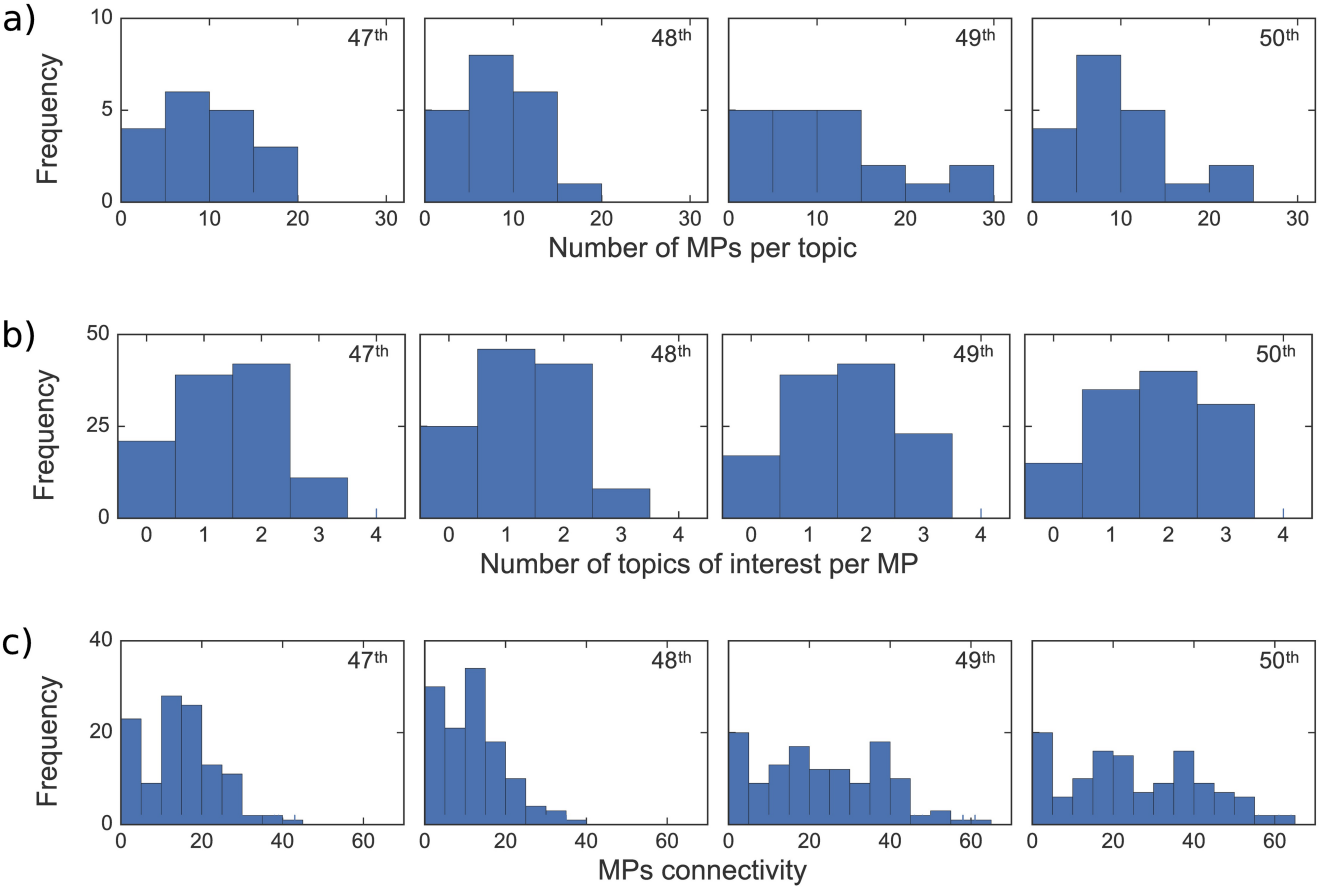
Degree distributions. **(a)** Number of MPs linked to each topic in the original bipartite network, **(b)** number of topics linked to each MP in the original bipartite network, and **(c)** number of links each MP has to other MPs in the MP-projected network.

The degree distributions in Fig 9 also tell an interesting story. From Fig 9a we see that topics are attracting the interest of more MPs over time. Associate with that is Fig 9b that shows the distribution of the number of topics that MPs spend the most time on during each government. Most MPs speak about 1-2 topics with larger repertoires of topics appearing in the 50^th^ parliament, since it has more MPs speaking about three topics than in the previous parliaments.

From Fig 9c, we can see that, during the National government in the 49^th^ and 50^th^ parliaments, the degree distributions of the MPs networks become more uniform. The tendency towards more uniformly connected networks suggests that National MPs (which are the most abundant during the last terms) share more common interests among them than Labour party MPs did when they were in government. With the aid of Fig 7, it also suggests that National MPs concentrate their speeches in a small set of topics, while there is more diversity of shared subjects among MPs of the other parties. In Fig 7, the 49^th^ and 50^th^ parliaments show the formation of a well-defined National Party group. In particular, in the 50^th^ parliament, the members of the largest community are connected to at least 45 other MPs, most of whom are also members of this tightly interconnected National Party group.

**Fig 10:**
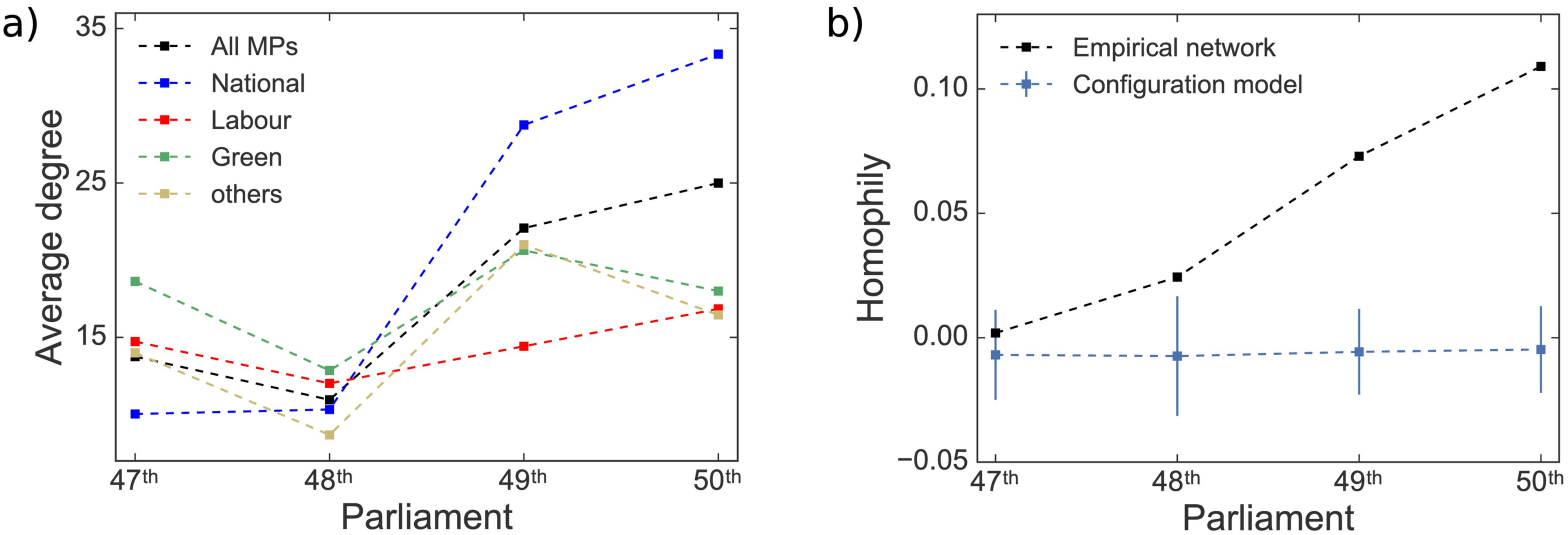
Average degree and homophily. **(a)** Time series of the average degree of MPs in the MP-projected network, decomposed by party. **(b)** Time series of net homophily in the MP-projected networks for the empirical data and configuration model. The results show average and standard deviation over 1000 runs.

Also supporting this idea of a close-knit community made up of National Party MPs is Fig 10b. It compares homophily between MPs—calculated using assortativity coefficient [13] for party affiliation—in the empirical networks with a configuration model [18, 19]. The configuration model is a random network model which preserves the degree sequence of the empirical network but has the links rewired. Therefore, for a configuration model, the expected homophily would be fairly constant, meaning that the party to which MPs belong would not influence connections between them. However, what we observe in Fig 10b for the empirical network is an increasing homophily, i.e. MPs preferentially share interests with other members of their own party. This is particularly true for the National Party.

## 4 Discussion

Topic models provide a way to parse human speech and extract themes from large bodies of text that are often difficult and time consuming to analyze manually. In few cases is it more important to gather and process this information than in the speeches of those people that control the legislative and political direction of a country. Topic modeling is unlikely to replace traditional media analysis of political speech, however, here we have shown that it can be a useful tool for examining the themes and trends in political discourse from a quantitative perspective.

We were able to use topic modeling to track changes in the content of parliamentary speeches across time, as well as to identify features in these time-series that correspond to particular events. In the time period examined, a number of large events influenced political discourse in New Zealand, such as the 2011 Christchurch earthquake, the global economic crisis and changes to local government with the creation of the Auckland *Super City* via the amalgamation of numerous smaller councils (see S1 Fig) as well as more recently, the housing crisis. Parliamentary discussions around all of these topics were identified, alongside more conventional themes such as the economy, the budget, and social welfare.

By breaking down these topics by time and party we have shown the different emphases that parties put on certain topics. For example, Fig 6 shows that much of the discussion around the developing housing crisis was pushed by Labour while the governing National Party showed little additional interest. Conversely, around the time of the economic downturn the National Party spent more time talking about economics. Unsurprisingly, the party that spent the most time discussing the environment was the Green Party. In other cases, such as the discourse surrounding the Christchurch earthquake and the governance of the Canterbury region (Fig 6), the increase in discussion was driven by all parties together. Important exogenous events, such as the Christchurch earthquake, force politicians from any political party to comment, shifting their discourse away from party lines. On the contrary, topics for which parties show major differences in trends suggest endogenous drivers. Therefore, the popularity of such topics is a direct result of conscious decisions made by political parties. For instance, the National Party’s apparent indifference to the increasingly vocal opposition parties’ discussion around housing, would suggest that National were consciously choosing not to engage with this topic. On the other hand, the Labour and Greens were deliberately choosing to talk about it during the period examined.

Although our analysis allows to distinguish between exogenous and endogenous drivers of debate, it can not always uncover the precise mechanisms affecting the level of discussion. For instance, the increase in the discussion of the economy by National party occurs at the same time as the global financial crisis, but it also coincides with the National Party taking power. Consequently, the change in discussion could be attributed to the crisis, or it could be that the governing party simply talks proportionally more about the economy when they first enter office. Studying a wider time window which includes several changes of government could help deciphering this sort of pattern.

Topic models also produce natural two-mode networks that can be decomposed into its projections and analyzed using standard complex network techniques. We have demonstrated that the resulting networks can display useful information such as community structure and interpretable degree distributions. Extracting and examining the top three communities for each parliament we showed how the National Party came to dominate discussion during the 49^th^ and 50^th^ parliaments. Moreover, we unveiled a gradual decline in the participation of smaller parties in these largest communities, in particular, having a minor influence in the largest community in the 50^th^ parliament.

Additionally, we detected (Fig 9) an increase in the number of topics of interest per MPs over time, indicating a decreasing topic specialization by individual MPs in their parliamentary speeches. This has resulted in MPs becoming more connected over time, specially those MPs belonging to the National party (Fig 10a). When considered in light of the aforementioned communities shown in Fig 8, our analysis suggests a widening of political discourse in New Zealand with opposition parties covering a greater number of topics, and the development of tight knit communities consisting of government MPs talking about a smaller range of topics.

The inner workings of many parliamentary systems may appear to be black-boxes to all but the most determined of citizens. The digitalization and accessibility of databases such as Hansard open the door to the empirical exploration of subjects which are critical to the function and prosperity of society. Our quantitative approach to the analysis of political discourse, based on a combination of methods from both topic modeling and complex networks, demonstrates the benefits of interdisciplinary research and suggests further interesting areas of study. For instance, annotating networks with demographic information would aid in the analysis of the role of gender, ethnicity, or educational background in national politics. Moreover, this approach could be used to investigate behavioral differences between ‘safe’ MPs near the top of their party list versus MPs who depend on electoral votes for re-election and elucidate the effects that local issues may have on politicians priorities. It is our hope that this work and others like it spur on further interdisciplinary research in order to explore these and any other possibilities yet to arise.

## Supporting information

S1 Table Codes and names of members of the parliament. Members of the Parliament and their respective codes in the networks. Codes that are missing in this table are due to the fact that some speakers do not show in the network. This is either because they were invited speakers (not a MP) or because the MP had spoken too few words (below ten thousand words) during the entire term.
(PDF)

S2 Table Topics identified and keywords. Topics identified and their respective key words from Mallet. *Topics that were removed from the networks. **Sometimes just part of Māori words appear due to accent marks. Those are not readable in the format used in Mallet (e.g. ‘whānau’ would appear as ‘wh’ only). Speeches in Māori are translated to English, i.e. the corpus in this work contains these speeches in their translated versions. Therefore, this topic needed to be removed from the network.
(PDF)

S1 Fig Time series of topic proportions. Time evolution of topic proportions, as discussed at Parliament and its decomposition by party.
(EPS)

S2 Fig Histogram of number of words. Frequency of number of words spoken by MP during the four parliaments.
(EPS)
