# Supplementary material for: Look who’s talking: Two-mode networks as representations of a topic model of New Zealand parliamentary speeches
Source: PLoS One. 2018 Jun 20;13(6):e0199072. doi: 10.1371/journal.pone.0199072 (PMC6010243; doi:10.1371/journal.pone.0199072)
Supplement: S1 Table — Members of the Parliament and their respective codes in the networks. Codes that are missing in this table are due to the fact that some speakers do not show in the network. This is either because they were invited speakers (not a MP) or because the MP had spoken too few words (below ten thousand words) during the entire term. (PDF) [file pone.0199072.s001.pdf]

Code	MP	Code	MP
1	Claudette Hauiti	118	Maurice Williamson
2	Larry Baldock	119	Doug Woolerton
3	Lesley Soper	120	Jian Yang
5	Keith Locke	121	Tariana Turia
6	John Hayes	122	Mahara Okeroa
7	Holly Walker	123	Gerry Brownlee
8	Pita Sharples	124	Craig McNair
9	Ian Mckelvie	125	Georgina Beyer
10	Ruth Dyson	126	David Shearer
11	Rod Donald	127	Marc Alexander
12	Paul Swain	128	Ron Mark
13	Jonathan Young	129	Jan Logie
14	Scott Simpson	130	Rodney Hide
15	Steve Chadwick	131	Nikki Kaye
16	David Clendon	132	Jacqui Dean
17	Pansy Wong	133	Cam Calder
18	Maggie Barry	134	Chester Borrows
19	Steven Joyce	135	Brendan Horan
20	Ashraf Choudhary	136	William Sio
21	Sue Moroney	137	Roger Sowry
22	Marian Hobbs	138	Nanaia Mahuta
23	Phil Heatley	139	Pita Paraone
24	Edwin Perry	140	Ken Shirley
25	Maryan Street	141	Chris Hipkins
26	Charles Chauvel	142	Roger Douglas
27	Rahui Katene	143	Allan Peachey
28	Michael Cullen	144	Alfred Ngaro
29	Georgina te Heuheu	145	Phil Twyford
30	Mike Sabin	146	John Tamihere
31	Richard Worth	147	Mike Ward
32	Sandra Goudie	148	Kate Wilkinson
33	Moana Mackey	149	Donna Awatere Huata
34	Andrew Little	150	Louise Upston
35	Julie-Anne Genter	151	Hone Harawira
36	Kevin Hague	152	Katherine Rich
37	Paul Foster-Bell	153	Paul Hutchison
38	Chris Tremain	154	Dave Hereora
39	Parekura Horomia	155	Judith Tizard
40	Nick Smith	156	Metiria Turei
41	Todd McClay	157	Jonathan Coleman
42	Sue Bradford	158	Simon Power
43	Damien O'Connor	159	Ann Hartley
44	Eugenie Sage	160	Stuart Nash
45	David Benson-Pope	161	Brent Catchpole
46	Asenati Lole-Taylor	162	Nathan Guy
47	Lindsay Tisch	163	Harry Duynhoven
48	Eric Roy	164	Denise Roche

49	Bernie Ogilvy	165	Kennedy Graham
50	Tau Henare	166	Lianne Dalziel
51	Richard Prosser	167	Helen Duncan
52	John Carter	168	Jim Peters
53	Jill Pettis	169	Andrew Williams
54	Lynda Scott	170	Carmel Sepuloni
55	Steve Maharey	171	Mark Gosche
56	Rajen Prasad	172	Catherine Delahunty
57	Nicky Wagner	173	Anne Tolley
58	Michael Woodhouse	174	Hekia Parata
59	Tim Macindoe	175	Jo Goodhew
60	Sam Lotu-Iiga	176	Te Ururoa Flavell
61	Richard Prebble	177	Matt Robson
62	Helen Clark	178	Simon Bridges
63	Tim Groser	179	Katrina Shanks
64	Amy Adams	180	Sue Kedgley
65	Rino Tirikatene	181	Taito Phillip Field
66	Darien Fenton	182	Bill Gudgeon
67	John Boscawen	183	Kelvin Davis
68	Bob Clarkson	184	Gareth Hughes
70	Gerrard Eckhoff	185	Trevor Mallard
71	Jonathan Hunt	186	Iain Lees-Galloway
72	Aaron Gilmore	187	Don Brash
73	Melissa Lee	188	Murray McCully
74	Tony Ryall	189	Pete Hodgson
75	Dianne Yates	190	Ross Robertson
76	Russel Norman	191	Jim Sutton
77	Poto Williams	192	Hilary Calvert
78	David Garrett	193	Jackie Blue
79	Phil Goff	194	Tracey Martin
80	Paul Quinn	195	Lynne Pillay
81	Heather Roy	196	Simon O'Connor
82	Martin Gallagher	197	Peter Dunne
83	David Clark	198	Lockwood Smith
84	Margaret Wilson	199	Darren Hughes
85	John Key	200	Chris Auchinvole
86	Grant Robertson	201	Rick Barker
87	Mark Burton	202	Winnie Laban
88	Clayton Cosgrove	203	George Hawkins
89	Jacinda Ardern	204	Brian Connell
90	Tim Barnett	205	Murray Smith
91	Clare Curran	206	Shane Ardern
92	Jim Anderton	207	Janet Mackey
93	Chris Carter	208	Mojo Mathers
94	Brendon Burns	209	Annette King
95	Nndor Tnczos	210	David Cunliffe
96	Deborah Coddington	211	David Parker
97	Kris Faafoi	212	Megan Woods
98	Mita Ririnui	213	Paula Bennett

99	Russell Fairbrother	214	Jami-Lee Ross
100	Kenneth Wang	215	Mark Blumsky
101	Wayne Mapp	216	Barbara Stewart
102	Carol Beaumont	217	John Banks
103	Muriel Newman	218	Mark Peck
104	Dail Jones	219	Kanwaljit Singh Bakshi
105	David Carter	220	Joanne Hayes
106	Gordon Copeland	221	Judy Turner
107	Christopher Finlayson	222	Steffan Browning
108	Brian Donnelly	223	Paul Goldsmith
109	Winston Peters	224	Craig Foss
110	Raymond Huo	225	Judith Collins
111	Denis O'Rourke	226	Colin King
112	Meka Whaitiri	227	Shane Jones
113	Dover Samuels	228	Stephen Franks
114	Mark Mitchell	229	Jeanette Fitzsimons
115	David Bennett	230	Peter Brown
116	Louisa Wall	231	Graham Kelly
117	Paul Adams		
