# Supplementary material for: Look who’s talking: Two-mode networks as representations of a topic model of New Zealand parliamentary speeches
Source: PLoS One. 2018 Jun 20;13(6):e0199072. doi: 10.1371/journal.pone.0199072 (PMC6010243; doi:10.1371/journal.pone.0199072)
Supplement: S2 Table — Topics identified and their respective key words from Mallet. *Topics that were removed from the networks. **Sometimes just part of Māori words appear due to accent marks. Those are not readable in the format used in Mallet (e.g. ‘whānau’ would appear as ‘wh’ only). Speeches in Māori are translated to English, i.e. the corpus in this work contains these speeches in their translated versions. Therefore, this topic needed to be removed from the network. (PDF) [file pone.0199072.s002.pdf]

**Topic**	**Key words**
Budget	government tax budget percent money zealanders labour national billion zealand cuts income year rate cut million superannuation spending rates english
Canterbury	volume date sitting text incorporated bound christchurch house page important good support piece time back work call part earthquake canterbury
Crime	police crime victims law prison justice person criminal people order offenders offence corrections offences community system officers prisoners bill parole
Drugs	bill people alcohol issue young age harm problem drugs legislation law make drug evidence tobacco support dog volume control smoking
Economy	government zealand economy economic labour jobs zealanders growth country development business plan world investment businesses sector national future years research
Education	education school schools students government national zealand funding training early tertiary science system teachers minister childhood student research standards television
Employment	work workers people labour employment bill day employers working compensation time pay minimum government wage acc employees hours business relations
Environment	environment management government energy emissions change water environmental climate resource conservation green national electricity zealand scheme trading oil marine carbon
Finance	zealand financial companies company bank assets finance business investment owned capital commerce market state banks sector money reserve power fund
Foreign affairs	zealand united international country people world countries defence foreign human rights immigration states force government australia security nations pacific convention
Government	information public government commission report service security office commissioner chief minister inquiry agencies general department executive review ministry authority independent
Growth	percent year years million government increase cost number increased costs rate time period significant level numbers increasing impact report total
Healthcare	health services care board king district people minister zealand public annette national ryall tony dr mental hospital government medical years
Housing	government housing building house people home homes state zealand property houses land problem auckland labour years national social affordable minister
Law and Order	law court rights legal justice case general act parliament made decision courts electoral evidence high cases judge matter person system
Legislation	bill act ensure provisions committee provide legislation regulatory review amendment regulations important current process time authority protection required regime move

Local government	local council government auckland city community people board councils bill communities canterbury area members trust district regional environment central authorities
Māori**	te ori ng ki iwi settlement ti treaty nei crown kia wh koutou land tou waitangi tau ora ko nau
Miscellaneous*	people things members back good thing put lot bit talk side sort time talking country fact thought heard absolutely happened
Political party*	party national labour members government john election vote parliament mr key leader house opposition policy political zealanders speech zealand don
Primary industries	zealand industry trade agreement country free farmers food primary dairy export world industries products biosecurity production china important market agreements
Procedural terms*	bill committee legislation select house process members time reading support important parliament submissions issue made stage issues debate forward bills
Procedural terms*	bill part act clause amendment legislation order paper supplementary section minister amendments committee title provisions purpose definition page provision call
Procedural terms*	mr member speaker order hon point house raise members mallard trevor chairperson assistant peters robertson interruption question debate brownlee winston
Procedural terms*	minister hon question prime dr answer mr david questions member asked smith government table statement document made house leave rt
Rhetoric*	people great time day house years parliament world today life country proud acknowledge history nation work place rugby team hard
Rhetoric*	issues green issue party point view deal debate society future problem fact make things simply terms approach sense process gambling
Social welfare	children people families work family social child young support benefit parents youth women care working poverty welfare vulnerable government leave
Tax	bill tax system scheme pay revenue money people department make income loan interest student levy support fair costs small time
Transport	transport government road public national auckland roads minister zealand land hon vehicle rail car people money infrastructure williamson charges bridges
